# Diamond Flap Anoplasty for Severe Post-hemorrhoidectomy Anal Stenosis: A Case Report

**DOI:** 10.7759/cureus.107038

**Published:** 2026-04-14

**Authors:** Stefan Dimov, Janja Konjevod, Ana Dimova, Martin Boroš, Lucija Grbić, Petra Vita Kasović

**Affiliations:** 1 Surgery, Zabok General Hospital and the Croatian Veterans Hospital, Zabok, HRV; 2 Surgery, University of Zagreb, School of Medicine, Zagreb, HRV

**Keywords:** anal sphincter, anoplasty, hemorrhoidectomy, rectal diseases, surgical flaps

## Abstract

Anal stenosis is an uncommon but important complication of anorectal surgery, most frequently occurring after hemorrhoidectomy. While mild cases may be managed conservatively, severe stenosis typically fails to respond to non-surgical interventions and requires operative correction. The diamond flap technique has increasingly been recognized as a valuable option, providing both optimal tissue perfusion and good functional outcomes.

We report the case of a 60-year-old man with a history of hemorrhoidectomy performed approximately 20 years earlier who presented with long-standing symptoms of obstructive defecation. After conservative management failed, surgical treatment was undertaken using a diamond-shaped mucocutaneous flap for scar excision, reconstruction of the anal canal, and limited external anal sphincter release. This case report describes a patient with severe anal stenosis developing after hemorrhoidectomy who was successfully treated with diamond flap anoplasty.

Diamond flap anoplasty is an effective surgical approach for the management of severe anal stenosis following hemorrhoidectomy. In this patient, the procedure achieved anatomical restoration and adequate widening of the anal canal while preserving sphincter integrity, resulting in a positive outcome. Awareness of anal stenosis as a potential late complication of hemorrhoidectomy is essential, as early recognition facilitates appropriate selection of reconstructive techniques and optimizes functional outcomes.

## Introduction

Anal stenosis is a rare yet disabling condition that causes significant discomfort. Patients commonly present with anal pain, difficult defecation, incomplete evacuation, and noticeably reduced stool caliber. Most patients will become habitual users of laxatives and enemas as a result of severe symptoms. The literature indicates that hemorrhoidectomy is responsible for approximately 90% of all anal stenosis cases, though it may also result from fissurectomy or fistulectomy, highlighting the importance of understanding its etiology and clinical implications [1,2].

Anal stenosis develops when the normal anoderm is replaced by dense fibrotic tissue, leading to a narrowed anal canal that lacks normal elasticity [3]. The stenotic segment may involve a partial circumference of the anal canal, or it may be diffuse and affect the entire anal canal [4]. Several classification systems exist for anal stenosis, with Khubchandani's method categorizing it as congenital, primary, or secondary [5]. Congenital stenosis typically stems from developmental anomalies like imperforate anus or anal atresia. In this classification, primary anal stenosis is categorized as involutional stenosis, a condition associated with senility. Most cases of anal stenosis are secondary, resulting from surgical trauma (e.g., hemorrhoidectomy, perianal excision, fistulectomy, sphincteroplasty), radiation therapy, perineal trauma, chronic suppuration, prolonged laxative use, Paget disease, or anal dysplasia [4,5]. As previously stated, hemorrhoidectomy is responsible for the majority of all anal stenosis cases. Given the high volume of surgical hemorrhoidectomies and the extensive anorectal mucosal trauma involved, hemorrhoidectomy is the leading cause of anal stenosis [1]. While modern surgical approaches aim to minimize complications, the literature documents that anal stenosis can still complicate radical amputative hemorrhoidectomy in up to 10% of cases [6-11]. This is also the most troublesome long-term complication following hemorrhoidectomy, which could be prevented with more conservative resection of the anoderm. Data shows stenosis is more common after emergency than after elective hemorrhoidectomy, highlighting the type of procedure used was found to be a significant factor in the development of stenosis [12].

Clinically, anal stenosis is classified into three groups according to anal examination with the Hill Ferguson (HF) retractor or index finger for mild and moderate cases, while small finger examination is related to severe cases [4]. The treatment of anal stricture depends on its position in the anal canal and its severity. Upper strictures are usually more difficult to treat than lower strictures, with mild stenosis usually responding to conservative therapy such as the use of stool bulk formers and dilatation [13]. Surgical management is reserved for patients with moderate to severe stenosis or those who have not responded to conservative treatment modalities. When selecting the appropriate surgical approach, it is crucial to assess if the anoderm pathology is also connected to the sphincter mechanism [3]. Surgical treatment for anal stenosis typically involves using various types of flaps for the reconstruction of the anal canal [14]. The diamond flap technique was first described in 1986 by Caplin and Kodner, and since then has been one of the preferred treatment options for moderate and severe anal stenosis [3]. 

Given the potentially disabling nature of symptoms and the technical complexity of surgical correction, early recognition of this complication and familiarity with available reconstructive options are essential for achieving optimal functional outcomes. The present case illustrates a severe, late-onset presentation of anal stenosis following hemorrhoidectomy successfully treated with diamond flap anoplasty and offers an opportunity to review this technique in light of contemporary scientific evidence.

## Case presentation

We present a case of a 60-year-old man complaining of a long-standing history of obstructive defecation symptoms that had gradually progressed over several years. He reported infrequent bowel movements occurring every few days, significant straining during defecation, and passage of narrow, ribbon-like stools. Regular use of laxatives provided minimal relief, and he frequently experienced a sensation of incomplete evacuation. His surgical history was notable for an open hemorrhoidectomy performed approximately 20 years earlier. There was no clinical history suggestive of inflammatory bowel disease, pelvic radiotherapy, or anorectal malignancy.

Physical examination revealed a markedly narrowed anal caliber measuring approximately 10 mm with dense, circumferential fibrosis at the anal verge, precluding digital rectal examination and consistent with severe cicatricial anal stenosis. In view of the severity of symptoms and failure of conservative management, surgical intervention was indicated.

**Figure 1:**
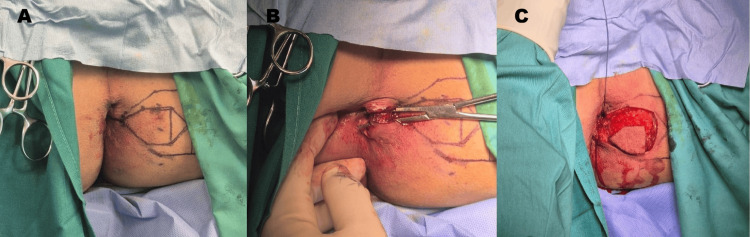
Intraoperative view and shaping of the diamond flap A: pre-incision intraoperative view illustrating the marking and planning of the diamond-shaped mucocutaneous flap adjacent to the stenotic anal canal; B: initial incision along the pre-marked diamond-shaped mucocutaneous flap; C: following complete excision of fibrotic tissue, a wide anal canal defect is revealed prior to flap advancement.

The procedure was performed under general anesthesia with the patient in the lithotomy position. Following adequate exposure and preparation of the operative field, careful preoperative planning of the reconstruction was undertaken. A diamond-shaped mucocutaneous flap was designed and marked adjacent to the stenotic anal canal to ensure appropriate dimensions and preservation of a reliable vascular pedicle (Figure 1A). Incisions were made along the pre-marked flap margins, followed by meticulous dissection through the subcutaneous tissue (Figure 1B). Dense fibrotic scar tissue responsible for the circumferential narrowing of the anal canal was identified and progressively excised. A limited release of the constricting fibrotic ring, including superficial fibers of the external sphincter, was performed with particular attention to preserving sphincter integrity and function (Figure 1C).

**Figure 2:**
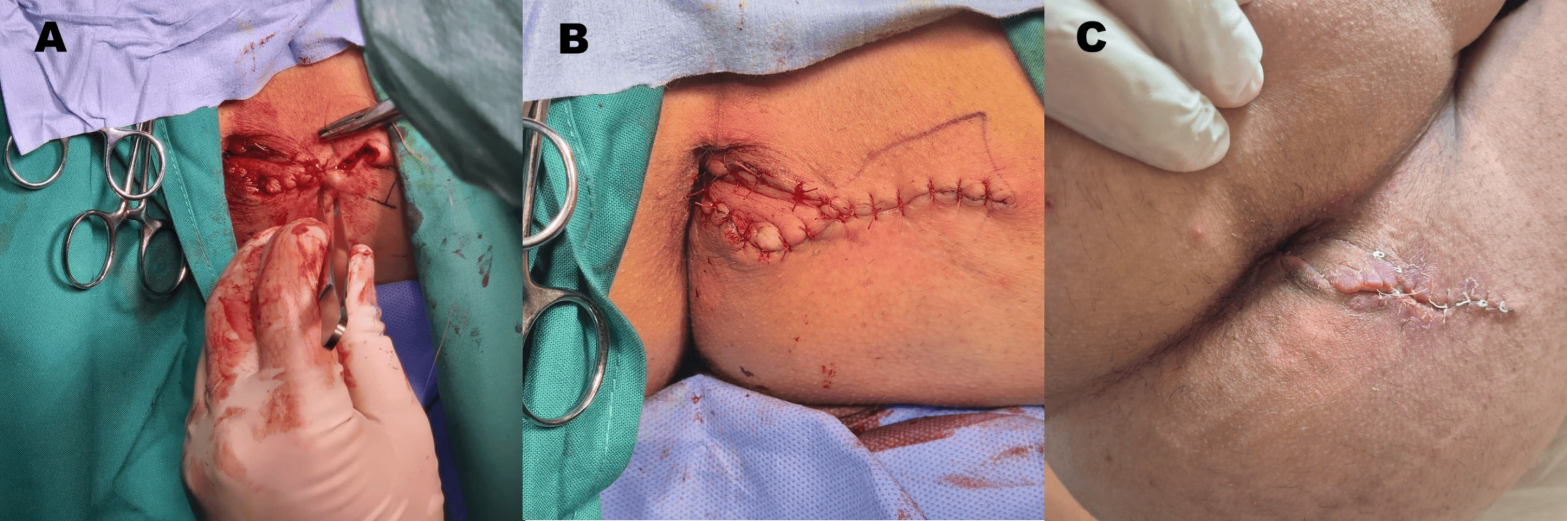
Diamond-shaped flap advancement, final positioning, and healed wound on follow-up appointment A: intraoperative view of diamond-shaped flap advancement, positioning, and suturing; B: final intraoperative view after diamond flap anoplasty, showing the advanced mucocutaneous flap sutured in a tension-free manner with adequate widening of the anal canal and tissue perfusion; C: postoperative follow-up view at two weeks demonstrating satisfactory wound healing, preserved perianal anatomy, and restored anal canal caliber.

After complete excision of the scar tissue, a wide defect within the anal canal was exposed, underscoring the severity of the stenosis and confirming the need for reconstructive anoplasty (Figure 1C). The diamond-shaped flap was then mobilized on its vascular pedicle and advanced into the defect (Figure 2A). Adequate perfusion was carefully assessed, and the flap was positioned without tension before fixation (Figure 2B). Interrupted absorbable sutures were used to secure the flap, resulting in immediate widening of the anal canal. Intraoperative digital examination confirmed satisfactory enlargement and improved tissue compliance. The final operative appearance demonstrated a flap with adequate perfusion with the restoration of normal anal caliber.

The postoperative course was uneventful. The patient reported significant improvement in defecation patterns, normalization of stool caliber, and complete resolution of obstructive symptoms. Follow-up examination at two weeks post-surgery showed appropriate wound healing, preserved perianal anatomy, normal sphincter tone, and no evidence of wound-related complications or restenosis (Figure 2C). At the six-month follow-up, index finger examination was possible, with an anal caliber measuring 25 mm. The patient reported no additional complications or adverse outcomes.

## Discussion

Anal stenosis is not uncommon after anal surgery, as its rate has been reported up to 10% in patients who have undergone hemorrhoidectomy [7-12]. Severe anal stenosis is defined as the inability of either a lubricated little finger or a small HF retractor to penetrate the anus [4]. Surgery is indicated in cases of severe anal stenosis. When selecting appropriate treatment, surgeons should assess the involvement of the anoderm as compared with the underlying sphincter mechanism [3]. The goal is to transfer either rectal mucosa or perianal skin to the anal canal to restore elasticity and to restore the diseased, nonpliable anoderm with elastic and compliant neoanoderm [3,15]. A procedure to bring in healthy perianal skin is not likely to help the patient with a stenotic muscle unless the patient has a concomitant sphincterotomy. A simple stricture release may provide temporary symptom relief but should be avoided, as it can lead to progressive scarring and subsequent stricture recurrence [3].

Regarding anoplasty for anal stenosis, multiple surgical techniques are available. The choice of procedure depends on both the severity of the pathology and the surgeon’s experience [2,16-18].

In our patients' case, we opted for the diamond flap anoplasty. The diamond flap anoplasty involves incising the scar tissue and leaving a diamond-shaped defect, with the diamond-shaped flap being created distally by incising to the subcutaneous fat. The apex and borders of the flap are sutured to the mucosa and surrounding anoderm of the intra-anal portion of the defect. This flap type offers straightforward construction, preserves flap vascularity, applies minimal tension to sutures, and allows primary donor site closure. A study on 18 consecutive patients with moderate to severe anal stenosis, who had undergone excisional hemorrhoidectomy previously, who underwent calibrated diamond anoplasty showed that using diamond advancement flaps to achieve an anal canal caliber of 25 to 26 mm resulted in favorable outcomes [19]. Another study conducted in 2022 showed that all patients with severe anal stenosis who underwent double diamond flap anoplasty achieved good outcomes, with their postoperative quality of life improving significantly [20].

Literature also describes other flap types, including the Y-V flap, usually reserved for stenoses below the dentate line [3]; the V-Y advancement flap anoplasty, originally developed for anal ectropion but applicable to anal stenosis [21]; the U-shaped flap that involves incision of scar tissue at the stenosis site without damaging the underlying sphincter [22]; the "house" flap anoplasty described by Christensen et al. as a V-Y flap modification for proximal stenoses or those extending from the dentate line to perianal skin [23]; and rotational S-plasty full-thickness flaps where S-shaped perianal skin is rotated around the central anal canal [15]. To date, no procedure has been conclusively proven superior, owing to the wide variability in disease severity and the diversity of surgical approaches, which makes it hard to conduct high-quality clinical trials [2].

It is also important to discuss strategies for preventing the development of anal strictures. In all perineal procedures, surgeons should avoid excessive undermining or removal of healthy anoderm and avoid injuring the sphincter muscles. During hemorrhoidectomy, which accounts for around 90% of anal stenosis, preserving viable tissue bridges is essential to promote optimal healing and minimize scarring [1,2,17].

## Conclusions

Anal stenosis following hemorrhoidectomy is a disabling condition that, depending on the severity of the stenosis, often requires surgical intervention. Diamond flap anoplasty offers a reliable reconstructive solution, restoring anal canal patency while preserving sphincter function and ensuring adequate tissue perfusion. The present case illustrates that this technique can lead to reliable functional improvement in advanced cicatricial stenosis. Additionally, it emphasizes the importance of surgical precision during the initial hemorrhoidectomy to reduce the risk of this challenging late-onset complication.
